# Rescue by Hypothermic Oxygenated Machine Perfusion for Unexpected Long Prolongation of Cold Preservation Time in Expanded Criteria Donor Kidneys Can Achieve Favorable 2‐Year Outcomes

**DOI:** 10.1111/ctr.70557

**Published:** 2026-05-08

**Authors:** Silvia Mingozzi, Alberto Mella, Caterina Dolla, Ester Gallo, Ana Maria Manzione, Enrico Sanna, Paolo Randone, Rita Tarragoni, Roberta Giraudi, Gloria Giovinazzo, Claudia Melloni, Aldo Verri, Andrea Bosio, Paolo Gontero, Antonella Barreca, Fabrizio Fop, Luigi Biancone

**Affiliations:** ^1^ Renal Transplant Center “A. Vercellone”,Division of Nephrology, Dialysis and Renal Transplant Division, Department of Medical Sciences “Città Della Salute e Della Scienza” Hospital, University of Turin, Italy Turin Italy; ^2^ Division of Vascular Surgery Department of Thoracic‐Vascular Surgery Città Della Salute e Della Scienza Hospital Turin Italy; ^3^ Division of Urology Department of Surgical Sciences AOU Città Della Salute e Della Scienza, Torino School of Medicine Turin Italy; ^4^ Pathology Unit University of Turin, Città Della Salute e Della Scienza Turin Italy

**Keywords:** cold preservation time, delayed graft function, kidney transplantation, oxygenated hypothermic machine perfusion

## Abstract

**Background:**

Hypothermic oxygenated machine perfusion (HOPE) is occasionally used to overcome logistical issues and allow longer cold preservation time (CPT); however, reports regarding these situations in older brain‐dead donors are lacking.

**Methods:**

Retrospective analysis of kidney transplants (KTs) performed between January 1, 2015, to December 31, 2023, from extended criteria donors (ECDs) switched to HOPE after a median time of 17.1 h in static cold storage (SCS) for unexpected logistic reasons and with a median total CPT of 28.6 h (25.3–32.0) (*n* = 44, HOPE ECDs), compared with a control group of KTs (*n* = 44, L‐ECDs) maintained in SCS alone with a low CPT (median, 10.4 h; range, 7.1–11.1) and matched for age, sex, donor age, donor eGFR, pre‐emptive transplant, and cPRA.

**Results:**

No primary nonfunction was observed, and both death‐censored graft survival (DCGS) and renal function progressively improved, remaining satisfactory and superimposable between groups up to two years post‐transplant (97.7% of DCGS with mean eGFR 46.5 mL/min/1.73 m^2^ [36–55.65] in HOPE‐ECDs) versus 97.7% with 42.0 (30.2–48.2) in L‐ECDs with similar KDPI (67% [49.75–86.5] vs. 72.5% [60.5–87.75], *p* = 0.475). These results were achieved in patients with HOPE‐ECDs, who experienced higher rates of DGF (31.8% vs. 11.4%, *p* = 0.036). No differences were noted in infection or rejection rates.

**Conclusion:**

Rescue by HOPE after SCS in ECDs with a very long CPT can achieve midterm outcomes similar to those with a low CPT in SCS alone, paving the way for its safe implementation in clinical practice.

**Abbreviations**
CPT: Cold preservation time
DGF: Delayed graft function
ECD(s): Expanded‐criteria donor(s)
HMP: Hypothermic machine perfusion
HOPE: Hypothermic oxygenated machine perfusion
KDPI: Kidney donor profile index
KT(s): Kidney Transplant(s)
KTR(s): Kidney Transplant recipient(s)
IQR: Interquartile Range
MMF: Mycophenolate mofetil
OR: Odds ratios
PNF: Primary non‐function
TAC: Tacrolimus
cPRA: calculated Panel Reactive Antibodies

## 1 Introduction

The disparity between the demand for kidney transplants (KTs) and the shortage of suitable organs has caused the number of patients awaiting KTs to progressively increase [1]. Expanded‐criteria donors (ECDs) have significantly and successfully increased the donor pool [2, 3], despite the potential risk of delayed graft function (DGF) with reduced graft survival compared with living and standard deceased donors; however, all of these aspects are overwhelmed by mortality in patients maintained on dialysis, stressing the importance and necessity of early transplantation in eligible patients [4].

DGF was also independently related to reduced outcomes, and although a clear cutoff value was not univocally stated, increased cold preservation time (CPT) was a determinant of increased DGF [5, 6, 7].

In this setting, machine perfusion, especially with hypothermic oxygenation techniques, is now considered a promising strategy for organ preservation, especially in donation after cardiac death [8, 9]. It may also prolong CPT, particularly in logistic settings where transplants cannot be easily performed [10, 11, 12, 13].

Although ECD organs are at high risk of developing DGF and could theoretically benefit from this approach, reports are limited. Real‐life operational situations may reveal the need for machine perfusion. This is the case of an unexpectedly prolonged CPT, especially when it occurs in ECD kidneys. Due to the nature of the topic, randomized clinical trials are impractical and unfeasible in this setting, and real‐life experiences are of paramount importance.

Therefore, the present study evaluated the outcomes in KT recipients (KTRs) who received a graft from an ECD with a very long CPT, using hypothermic oxygenated machine perfusion (HOPE) as a rescue, compared with a control group receiving CPT and static cold storage (SCS) at our high‐volume kidney transplantation center.

## 2 Materials and Methods

### 2.1 Studied Population and Outcomes

Clinical and renal functional data were extracted from the medical charts of all KT patients transplanted at the Turin University Renal Transplant Center “A. Vercellone” from January 2015 to December 2023. Each recipient was followed by a transplant center with at least one annual visit and by a local nephrologist (11 peripheral centers covering most of the Piedmont region) for periodic follow‐up.

**FIGURE 1:**
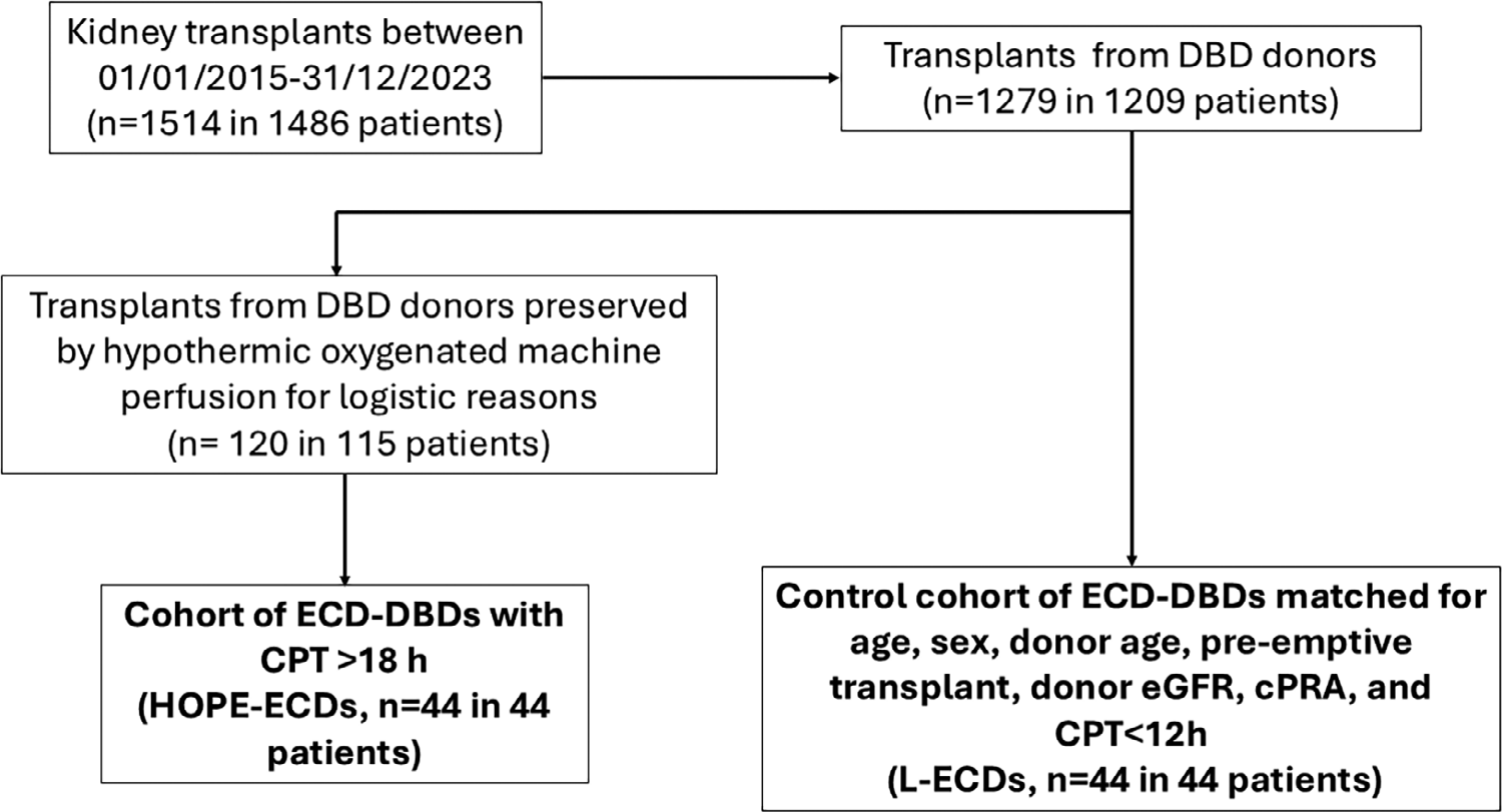
Flow chart and graphical schematization of the studied population. DCD, donation after circulatory death; DBD, donation after brain death; ECD, expanded‐criteria donors.

Therefore, we analyzed patients who received a graft from a beating‐heart ECD according to Crystal City criteria [14] and with long CPT (> 18 h) initially in SCS and switched to HOPE (Kidney Assist Device, Organ Assist BV, Groningen, the Netherlands), with genuine active oxygenation for unexpected logistic reasons (termed as HOPE‐ECDs). These patients were matched for age, sex, donor age, donor eGFR, pre‐emptive transplant, cPRA with KTRs receiving an organ with low CPT (< 12 h) preserved in SCS (low‐ECDs [L‐ECDs]) (Figure 1).

All organs were stored in SCS (UW Solution) during transportation from the organ procurement hospital to our transplant center, and HOPE was initiated thereafter (in these cases, CPT was the sum of SCS and HOPE). HOPE‐ECDs and all KTRs with an organ managed with HOPE, received a broad‐spectrum antimicrobial therapy with piperacillin/tazobactam and amphotericin B for at least 1 week, per local protocol.

Standard immunosuppression included induction therapy with basiliximab (Simulect; Novartis Pharmaceuticals Corp., East Hanover, NJ, USA) or rabbit anti‐thymocyte globulin (rATG; Thymoglobulin; Genzyme, Cambridge, MA, USA; dose 1.5–4 mg/kg) in association with steroids, according to recipient characteristics (i.e., immunological risk), and maintenance therapy composed of tacrolimus (TAC; level 10–12 ng/mL before three month post‐transplant, 8–10 ng/mL between 3 and 6 months post‐transplant, 6–8 ng/mL after 6 months), mycophenolate mofetil/mycophenolic acid and steroids. Antimicrobial and CMV prophylaxis were administered post‐KT according to donor/recipient serological status and induction therapy. All patients received prophylaxis against *Pneumocystis jiroveci* pneumonia with sulfamethoxazole–trimethoprim for six months and valganciclovir for CMV prophylaxis based on their individual risks and induction therapy.

Since June 2018, all patients who experienced prolonged DGF (i.e., the need for hemodialysis seven days post‐transplant) with oliguria/anuria and renal allograft biopsy with extensive acute tubular injury/severe calcineurin inhibitors nephrotoxicity could be switched to belatacept‐based immunosuppression [15] on clinical judgment, excluding KTRs with seronegative EBV status or active infections.

The primary outcome was death‐censored graft survival, and the secondary outcomes included DGF (defined as the need for dialysis within the first post‐transplantation week), graft rejection (biopsy‐proven), early (occurring during the hospital stay), late infection rates, and renal functional data.

This study was conducted in accordance with the latest version of the Declaration of Helsinki and the Principles of the Declaration of Istanbul. This study was approved by our institutional ethics committee (Approval No. 1449/2019; Approval date: August 11, 2019).

### 2.2 Statistical Analysis

Continuous variables were described using the median and interquartile range (IQR) due to their nonnormal distribution. The Mann‐Whitney *U*‐test was used to compare independent groups, and the Wilcoxon signed‐rank test was used to compare related variables.

Categorical variables are presented as fractions and the Pearson's correlation coefficient or, for small samples, Fisher's exact tests were employed to compare groups. Odds ratios (ORs) with 95% confidence intervals (CIs) were used as measures of relative risk (RR).

A control cohort was selected using stratified sampling and adjusted for age, sex, and renal function at the time of transplantation.

Univariate survival analysis was performed using the Kaplan–Meier method, and the log‐rank test was used to compare strata.

The significance level for all tests was set at α < 0.05.

All statistical analyses were performed using SPSS (IBM Corp., released 2021, IBM SPSS Statistics for Windows version 26.0, IBM Corp., Armonk, NY, USA).

## 3 Results

### 3.1 Population Characteristics and Graft Survival

The main characteristics of the studied population are included in Table 1.

**TABLE 1:** Baseline and post‐transplant clinical data of the studied population.

	HOPE‐ECDs with long CIT (*n* = 44)	L ‐ECDs (*n* = 44)	*p*‐value
**Recipients**			
Age at transplant (yrs), *median* (IQR)	59.5 (50–67.75)	62.0 (52.8–68.8)	0.314
Gender male, *n* (%)	30 (68.2)	31 (70.5)	1.00
Re‐transplantation, *n* (%)	7 (15.9)	8 (18.2)	1.00
Pre‐emptive transplantation, *n* (%)	4 (9.1)	5 (11.4)	1.00
Double kidney transplant, *n* (%)	6 (13.6)	1 (2.3)	0.110
Kidney disease, *n* (%)			
CKD (not otherwise specified)	10 (22.7)	14 (31.8)	
Diabetic nephropathy	3 (6.8)	4 (9.1)	0.680
Glomerulonephritis	7 (15.9)	9 (20.5)	
Polycystic kidney disease	9 (20.5)	9 (20.5)	
Urinary tract malformation/urological disease	6 (13.6)	4 (9.1)	
Other disease	9 (20.5)	4 (9.1)	
cPRA (%), *median* (IQR)	0 (0–0)	0 (0–45.9)	0.302
**Donors**			
Age (yrs), *median* (IQR)	65 (60–74.5)	65 (62–72)	0.741
eGFR, *ml/min/1.73m* ^2^ (IQR)	76.5 (56.25‐100.425)	83.2 (66.4‐109,5)	0.175
Total cold preservation time, *hours* (IQR)	28.6 (25.3‐32.0)	10.5 (7.1‐11.1)	**<0.001**
Hypothermic machine perfusion time, *hours* (IQR)	11.5 (6.25‐12.9)	NA	
KDPI, *%* (IQR)	67 (49.75–86.5)	72.5 (60.5–87.75)	0.475
**Post‐transplant**			
DGF, *n* (%)	14 (31.8)	5 (11.4)	**0.036**
DGF time, days (IQR)	14 (7.5–19.75)	8.5 (1.25–29.25)	0.497
PNF, *n* (%)	0 (0)	0 (0)	1.000
Rejection episodes, *n* (%)	5 (11.4)	4 (9.1)	1.000
DSA, *n* (%)	2 (4.5)	8(18.2)	0.089
Infection during hospital stay, *n* (%)	9 (20.5)	8 (18.2)	1.000
Infection during the f/up, *n* (%)	31 (70.5)	26 (61.9)	0.495
Length of hospital stay, *days* (IQR)	26 (16.25–34)	17 (13–25.5)	**0.042**
Immunosuppressive therapy			
Thymoglobulin, *n* (%)	37 (84.1)	29 (65.9)	0.084
Basiliximab, *n* (%)	7 (15.9)	15 (34.1)	
Tacrolimus, *n* (%)	42 (95.5)	44 (100)	0.494
Mycophenolate mofetil, *n* (%)	27 (61.4)	29 (65.9)	0.825
Steroids, *n* (%)	35 (79.5)	36 (81.8)	1.000
Belatacept, *n* (%)	4 (9.1)	9 (20.4)	0.229

*Note:* Significant *p*‐values are reported in bold.

Abbreviations: cPRA, calculated panel reactive antibody; DGF, delayed graft function; DSA, donor specific antibodies; eGFR, estimated glomerular filtration rate; IQR, interquartile range; NA, not applicable; PNF, primary non‐function.

As graphically summarized in Figure 1, among the 1,514 KTs performed involving 1,486 patients, we analyzed 44 KT patients in the HOPE‐ECD group, matched to 44 L‐ECDs for age, sex, donor age, donor eGFR, pre‐emptive transplant, and cPRA.

During the studied period, seven kidneys were perfused but not transplanted because Karpinski score ≥ 7 or macroscopic anatomic anomalies were revealed prior to transplantation. However, two kidneys exhibited abnormal renal resistances (> 0.8 mmHg/mL/min) during perfusion and were discarded. Renal biopsy revealed thrombotic microangiopathy and an elevated Karpinski score of 7, with a vascular score of 3, confirming severe vascular damage.

Based on patient selection criteria, KDPI scoring reflects their characteristics with similar values among groups (67% [49.75–86.5] in HOPE‐ECDs vs. 72.5% [60.5–87.75] in the L‐ECDs; *p* = 0.475), and CPT was significantly shorter in the L‐ECDs (median, 10.5 h [7.1–11.1] vs. 28.6 [25.3–32.0]; *p* < 0.001).

**FIGURE 2:**
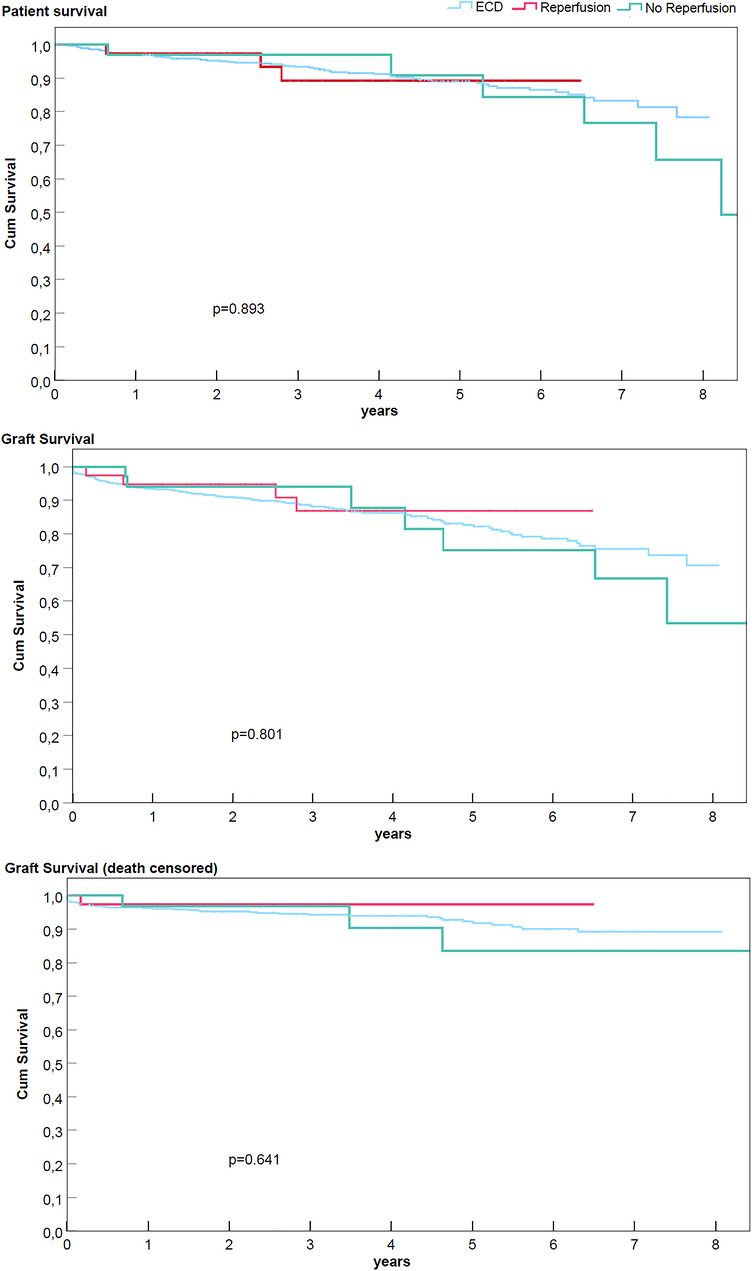
Patient and graft survival in the studied population. Patients in the HOPE‐ECDs group (Reperfusion) had similar patient and graft survival rates to those receiving organs maintained in static cold storage with low cold preservation times (No Reperfusion) and ECDs performed in the same period (ECD). HOPE, hypothermic oxygenated machine perfusion; ECD, expanded‐criteria donors.

Regarding primary outcomes, patient and graft survival rates were superimposable and satisfactory in both groups (97.7% death‐censored graft survival at 2 years in HOPE‐ECDs vs. 97.7% in L‐ECDs), compared with all ECDs performed in the analyzed period (Figure 2), excluding dual kidneys in each group (Figure S1), and considering a random group of recipients of standard‐criteria organs (Figure S2).

No patients in the HOPE‐ECD group experienced primary nonfunction (PNF).

### 3.2 Kidney Functional Tests and Post‐Transplant Outcomes

HOPE‐ECD patients experienced more frequent DGF (31.8% vs. 11.4%, *p* = 0.036) and a trend toward longer hospital stay. No significant differences in infection (during hospitalization or post‐transplantation period), rejection rates, or positive *de novo* donor‐specific antibodies were observed.

Additionally, patients in both groups were preferentially treated with thymoglobulin, TAC, mycophenolate mofetil (MMF), and steroids, with MMF/steroids tapered according to patient characteristics. Belatacept use was similar across the groups. Prolonged CPT, increased risk of rejection during the initial transplantation period, and the target to minimize calcineurin inhibitor nephrotoxicity in the early post‐transplant period may drive the trend toward the increased use of thymoglobulin in HOPE‐ECDs (Table 1).

**FIGURE 3:**
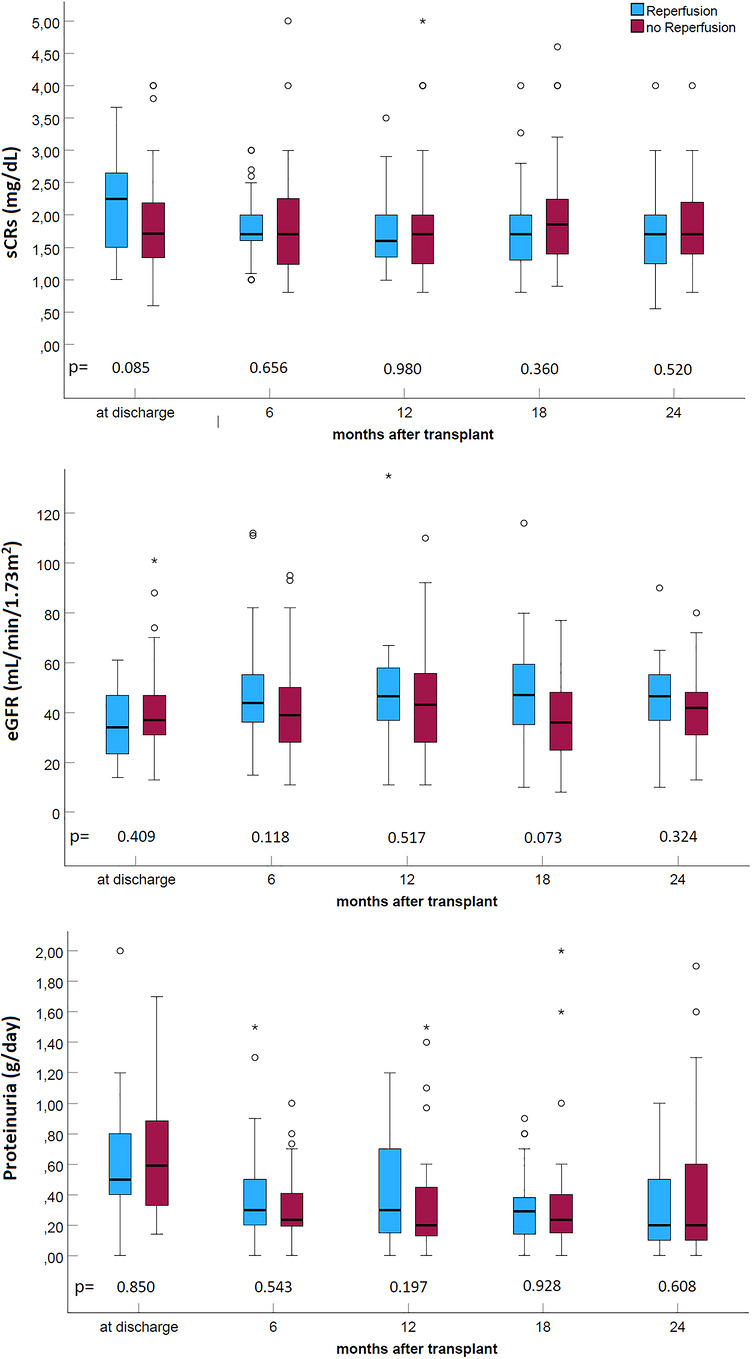
Kidney functional tests and proteinuria in the studied population.

Despite an initial trend towards reduced eGFR at discharge, patients in both groups exhibited similar eGFR and proteinuria values at all follow‐up visits (Figure 3).

## 4 Discussion

Hypothermic machine perfusion (HMP) has been intensively studied and has provided interesting results regarding DGF, acute rejection rates, and graft survival [16, 17]. In particular, a recent Cochrane meta‐analysis showed that all studies performed in the last decade documented a reduced incidence of DGF (RR, 0.77; 95% CI, 0.66–0.91; *p* = 0.002) and a critical cost‐effective advantage in both North American and European settings with HMP use compared to SCS [17]. These could be even more critical when ECD, which are constitutively more prone to DGF, were considered because positive benefits may determine increased feasibility in organ allocation, transplant outcomes, and costs [4].

Recent retrospective analyses have reported favorable outcomes for HMP in KTs performed using ECDs with prolonged CPT. Adani et al. retrospectively evaluated 73 versus 81 KTs managed with HMP and SCS, respectively, with median CPTs of 29 h and 57 min versus 11 h and 25 min, and an ECD prevalence of 43.8% versus 18.5%, respectively (*p* < 0.001). After adjusting for these two characteristics using a propensity score model, KTs showed comparable outcomes in terms of DGF, vascular, and urologic complications, infections, episodes of graft rejection, and kidney function at 1 year [18]. Foucher et al., in a more extensive matched cohort analysis with longer follow‐up, confirmed a significant reduction in the confounder‐adjusted percentages of DGF (24.0% in the HMP group vs. 32.2% in SCS) but with similar long‐term outcomes (mean life expectancy with functioning graft, 5.7 vs. 6 years) without any differences for patients receiving an organ from donors aged ≥ 70 years or those with a CPT ≥ 18 h [19].

The implementation of HOPE showed promising results in donation after circulatory death without a significant difference in ECDs treated with HOPE or SCS, although similar and relatively short CPT was compared (13.2 [5.1–28.7] vs. 12.9 [4–29.2]) [20].

The role of HOPE in high‐risk grafts was investigated in a recent post hoc analysis, which demonstrated lower DGF rates in donors > 60 years, albeit with a shorter CPT (13 h) [21].

As Hunter et al. recently noted, whether a prolonged period of HOPE following SCS would confer clinical benefits remains unclear, as does whether extending the CPT would worsen the overall injury [22]. Simultaneously, the supply of oxygen may be critical in older donors, those with acute kidney injury, and in grafts derived from donors with underlying medical comorbidities, such as hypertension, diabetes, and cardiovascular disease.

We aimed to assess whether HOPE could maintain successful and satisfactory outcomes in a real‐world setting, as in Adani et al. [18]., where the KT could be postponed for logistical reasons, and the CPT needed to be significantly prolonged with a very high risk of DGF and PNF [5].

In our matched‐case analysis, HOPE appeared to be a feasible option, maintaining both early‐ and mid‐term transplant outcomes (no PNF, low rates of transplant rejection, good death‐censored graft survival, and kidney function at 2 years) despite the very long CPT in HOPE‐ECDs. Comparator group was chosen based on stratified sampling. Additionally, adjustment was done for age, sex, donor age, donor eGFR, pre‐emptive transplant, and cPRA with KTRs receiving an organ with low CPT (< 12 h) preserved in SCS L‐ECDs. As this period of time in SCS was supposed to occur before unexpected logistic reasons, the plan in the case group was changed. We could show that this rescue strategy did not influence the two‐year graft survival or function of ECD kidneys. A control group with a longer CPT, which may be more representative of the average CPT in our center, could have exhibited lesser differences in DGF. However, this was not done as we intended to remain as close as possible to the comparison profiles of real‐life cases rescued by HOPE. Moreover, comparison with SCS‐only ECD kidneys that had very long CPTs (average 28 h, maximum 32 h), which has not been previously pursued, was not done. Indeed, this typical “plan B” operational situation, such as in the cases cohort, began to appear a few years ago, when our center steadily doubled the number of KTs, coincided with the beginning of local machine perfusion experience. In this study, we focused on ECDs because they represent the majority of donors and are more sensitive to CPT.

Longer hospital stays than reported in other studies was observed. However, these data need to be contextualized based on the overall complexity of our recipients, reported to be the highest in our country, according to data published by the Italian National Transplant Center, with the median age of enlisted patients among the highest in Italy [23].

Our study has some limitations due to its observational design. However, the specific setting of CPT prolongation could be considered a clear example of a nonethical randomized clinical trial in agreement with Foucher et al. Such trials are performed under optimal circumstances, resulting in nonrepresentative conclusions for clinical practice [19, 24]. For example, the number of cases per group required to achieve a power of at least 0.8 (to demonstrate that no clinically relevant difference in the predicted outcome) exceeded the number of patients who underwent HOPE in Italy during the study period.

In conclusion, we report an exploratory matched case analysis documenting the potential positive impact of rescue HOPE in maintaining good clinical and transplantation outcomes in KTs performed using ECDs with prolonged CPT. These findings should be confirmed through more extensive follow‐up studies; however, it may pave the way for implementing HOPE in the ECD setting.

## Author Contributions

Concept design, data collection, analysis, interpretation, and drafting: Silvia Mingozzi and Alberto Mella. Data collection and drafting: Caterina Dolla, Ester Gallo, Ana Maria Manzione, Enrico Sanna, Paolo Randone, Rita Tarragoni, Roberta Giraudi, and Gloria Giovinazzo. Critical revision: Claudia Melloni, Aldo Verri, Andrea Bosio, Paolo Gontero, and Antonella Barreca. Concept design, analysis, interpretation, drafting, and critical revision: Luigi Biancone. All authors provided intellectual content of critical importance to the work described and have approved the final version.

## Funding

The study was supported by the “TGT study” (University of Turin, Department of Medical Sciences) and “CRT foundation” grants to LB.

## Conflicts of Interest

The authors declare no conflicts of interest.

## Supporting information




**Supporting Information**: ctr70557‐sup‐0001‐figureS1‐S2.docx

## Data Availability

The data supporting the findings of this study are available from the corresponding author upon reasonable request.
