# Supplementary material for: Rescue by Hypothermic Oxygenated Machine Perfusion for Unexpected Long Prolongation of Cold Preservation Time in Expanded Criteria Donor Kidneys Can Achieve Favorable 2‐Year Outcomes
Source: Clin Transplant. 2026 May 8;40:e70557. doi: 10.1111/ctr.70557 (PMC13156423; doi:10.1111/ctr.70557)

**Supplementary Figure 1. Patient and graft survival in the studied population, excluding recipients of dual kidneys.** Patients in the HOPE-ECDs group (Reperfusion) have similar patient and graft survival rates to those receiving organs maintained in static cold storage with low cold preservation times (No Reperfusion).

**
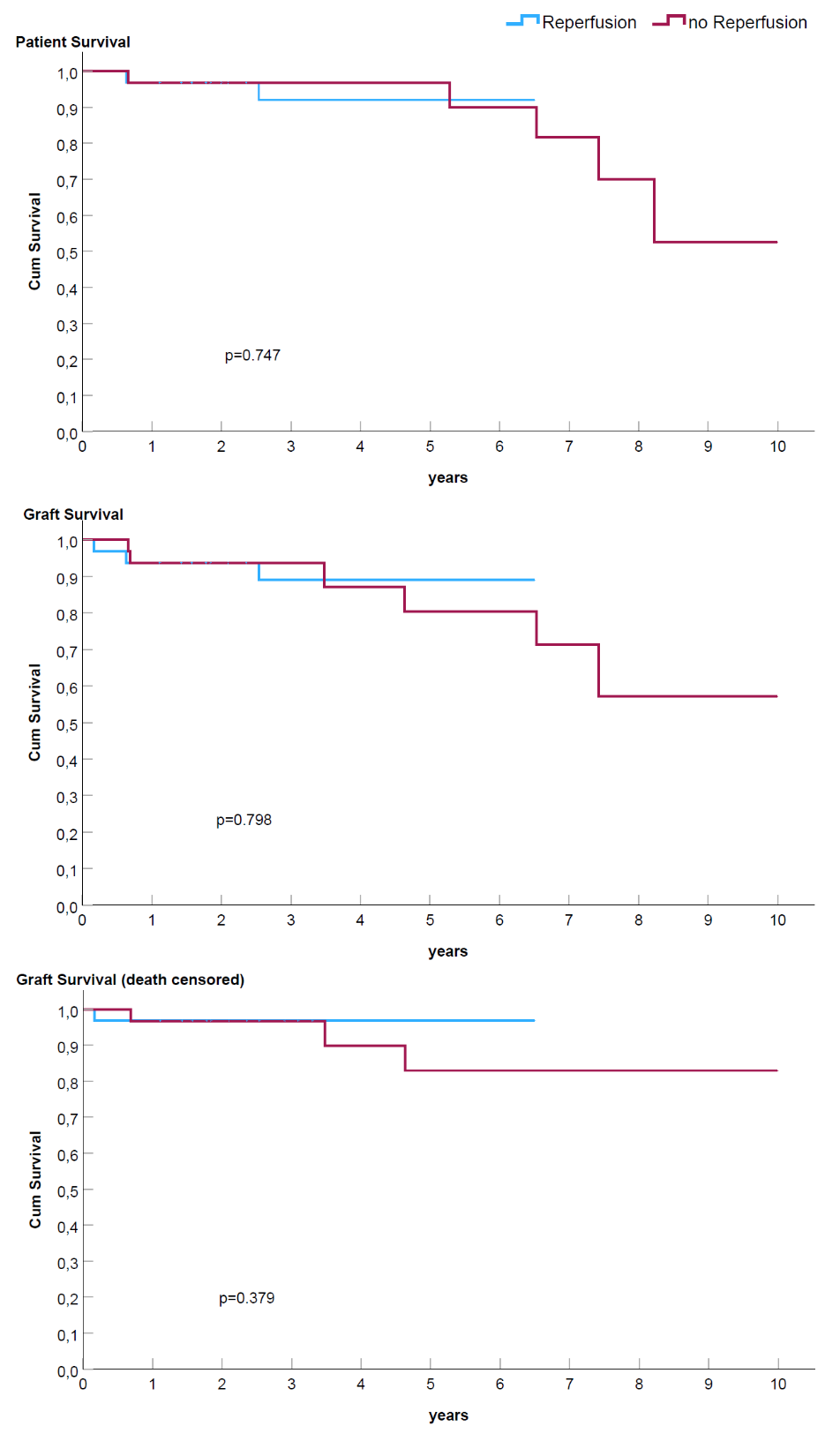
**

**Supplementary Figure 2. Patient and graft survival in the studied population, compared to a random group of standard-criteria organs.** Patients in the HOPE-ECDs group (Reperfusion) and those receiving organs maintained in static cold storage with low cold preservation times (No Reperfusion) have similar patient and graft survival rates to a random group of recipients of standard-criteria organs performed in the same period (SCS).


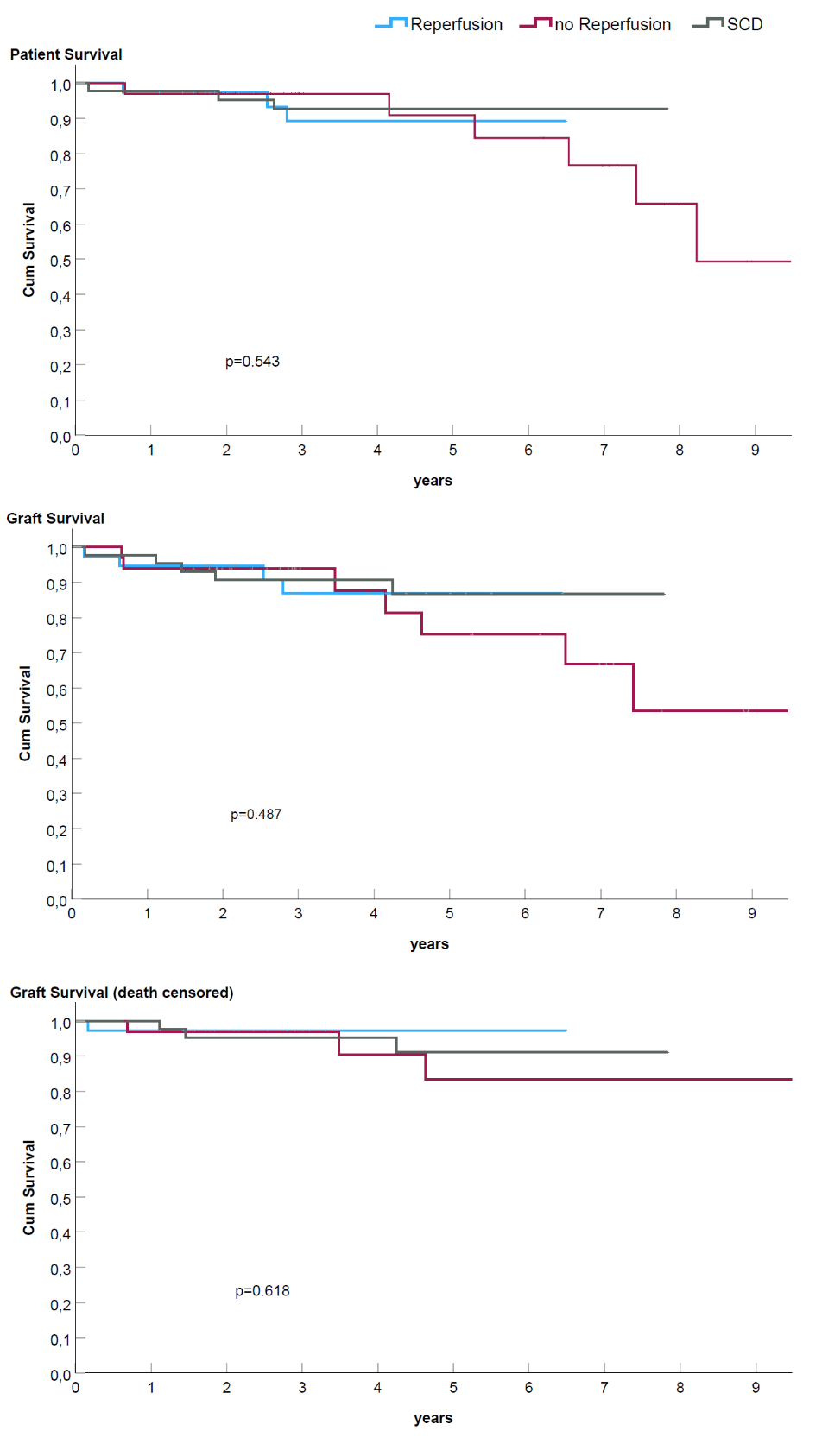

Supplement: Supplementary file 1 — Supporting Information: ctr70557‐sup‐0001‐figureS1‐S2.docx [file CTR-40-e70557-s001.docx]
